# Filling evidence gaps on the impact of pneumococcal vaccines

**DOI:** 10.1016/S1473-3099(17)30328-6

**Published:** 2017-06-07

**Authors:** Daniel M Weinberger

**Affiliations:** 1 Department of Epidemiology of Microbial Diseases, Yale School of Public Health, New Haven, CT

Pneumococcal conjugate vaccines (PCVs) are now widely used for children around the world. Because of the high cost of the vaccine, donor organisations, such as GAVI, The Vaccine Alliance, have developed innovative financing schemes to encourage their introduction in low-income countries. Given these efforts, it is essential to quantify the effect of the vaccines on disease rates. In The Lancet Infectious Diseases, Grant Mackenzie and colleagues^1^ use data from a population-based surveillance study in The Gambia to show the important impact of PCV7/13 against pneumonia. This study fills a crucial gap in our understanding of the effects of PCVs in low-income settings.

The effects of PCVs on invasive pneumococcal disease and pneumonia have been well documented, with data from randomised controlled trials and observational studies showing a consistent benefit across different epidemiological settings.^2–4^ However, relatively few data are available on the population-level impact of routine PCV use in Africa, where the burden of disease is among the highest in the world. Although several studies have reported a benefit from the vaccine for invasive pneumococcal disease,^5,6^ most of the burden of disease caused by pneumococcus is thought to be attributable to pneumonia.^7^ Therefore, evaluations of the effect of PCVs against pneumonia in Africa are crucial for understanding the overall impact of the vaccine.

The study by MacKenzie and colleagues has several notable strengths. The data on pneumonia are drawn from a well characterised population-based surveillance system, allowing for accurate estimates of incidence rates over time. Moreover, detailed clinical characteristics of the cases were known, allowing for evaluations of impact against several different definitions of pneumonia that vary in severity and specificity for pneumococcus. The study is also unusual in that the investigators did both an analysis of changes in diseases rates and a case-control analysis in the same population. Together, these analyses provide complementary pieces of evidence, with the analysis of disease rates providing an estimate of the population-level effect of the vaccine (direct and indirect effects) and the case-control analysis providing an estimate of direct effectiveness. The conclusions of the study are also strengthened by the inclusion of pre-specified sensitivity analyses, well justified control conditions, and detailed data on referral and testing patterns over time.

There are also notable caveats to the interpretation of the results from this study. While the vaccine had an impressive effect on laboratory-confirmed pneumococcal pneumonia, radiologically-confirmed pneumonia, and hypoxic pneumonia, the relative declines in the more common clinical pneumonia were modest (0–15% decline, depending on the definition). This result is expected because clinical pneumonia is the least specific definition.^8^ However, this finding highlights the large remaining burden of pneumonia caused by other pathogens that needs to be addressed with other interventions. Additionally, the analysis was limited by methodological challenges. The short pre-vaccine baseline period (<2 years) made it impossible for the investigators to do a formal time series analysis, which could be used to detect and adjust for underlying secular trends in pneumonia rates—the simple pre-vaccine vs post-vaccine comparison might improperly attribute unrelated declines to the vaccine.^9^ Finally, the results were sensitive to the use of a crude adjustment for changing referral patterns, which could potentially bias the results. Nonetheless, these data are among the best available evidence for the effects of PCVs on pneumonia in low-income settings.

These findings are likely to be impactful as WHO begins to review the evidence for the effects of PCVs against different outcomes, the effects of different PCV dosing schedules, and the indirect (herd) effects of different vaccines.^10^ Additional analyses of the trajectories of decline in different age groups in this population could help to resolve questions about the ability of a 3 + 0 dosing schedule (ie, three doses and no booster) to disrupt transmission and induce indirect protection in unvaccinated individuals.

MacKenzie and colleagues’ study presents an important set of estimates of the impact of PCVs against pneumonia in a low-income setting with a high burden of disease. This study can serve as a benchmark against which studies of PCV impact repeated in other countries in the region can be compared.
